# Hippocampal Dysfunction Provoked by Mercury Chloride Exposure: Evaluation of Cognitive Impairment, Oxidative Stress, Tissue Injury and Nature of Cell Death

**DOI:** 10.1155/2018/7878050

**Published:** 2018-04-10

**Authors:** Walessa Alana Bragança Aragão, Francisco Bruno Teixeira, Nathalia Carolina Fernandes Fagundes, Rafael Monteiro Fernandes, Luanna Melo Pereira Fernandes, Márcia Cristina Freitas da Silva, Lilian Lund Amado, Fernanda Espírito Santo Sagica, Edivaldo Herculano Correa Oliveira, Maria Elena Crespo-Lopez, Cristiane Socorro Ferraz Maia, Rafael Rodrigues Lima

**Affiliations:** ^1^Laboratory of Functional and Structural Biology, Institute of Biological Sciences, Federal University of Pará, Belém, PA, Brazil; ^2^Laboratory of Pharmacology of Inflammation and Behavior, Institute of Health Sciences, Federal University of Pará, Belém, PA, Brazil; ^3^Laboratory of Ecotoxicology, Institute of Biological Sciences, Federal University of Pará, Belém, PA, Brazil; ^4^Laboratory of Tissue Culture and Cytogenetics, Evandro Chagas Institute, Ananindeua, PA, Brazil; ^5^Laboratory of Molecular Pharmacology, Institute of Biological Sciences, Federal University of Pará, Belém, PA, Brazil

## Abstract

Mercury (Hg) is a highly toxic metal, which can be found in its inorganic form in the environment. This form presents lower liposolubility and lower absorption in the body. In order to elucidate the possible toxicity of inorganic Hg in the hippocampus, we investigated the potential of low doses of mercury chloride (HgCl_2_) to promote hippocampal dysfunction by employing a chronic exposure model. For this, 56 rats were exposed to HgCl_2_ (0.375 mg/kg/day) via the oral route for 45 days. After the exposure period, the animals were submitted to the cognitive test of fear memory. The hippocampus was collected for the measurement of total Hg levels, analysis of oxidative stress, and evaluation of cytotoxicity, apoptosis, and tissue injury. It was observed that chronic exposure to inorganic Hg promotes an increase in mercury levels in this region and damage to short- and long-term memory. Furthermore, we found that this exposure model provoked oxidative stress, which led to cytotoxicity and cell death by apoptosis, affecting astrocytes and neurons in the hippocampus. Our study demonstrated that inorganic Hg, even with its low liposolubility, is able to produce deleterious effects in the central nervous system, resulting in cognitive impairment and hippocampal damage when administered for a long time at low doses in rats.

## 1. Introduction

Mercury (Hg) represents the third most toxic element on the planet, according to the US Government Agency for Toxic Substances and Disease Registry [1]. Three forms of Hg can occur in the environment: organic Hg, elemental Hg, and inorganic Hg [2].

Organic Hg is one of the most toxic substances, with the highest number of studies dealing with their effects on human health [2–5]. It is known that organic Hg has a high capacity to cross cellular membranes. After ingestion, it can pass through the blood-brain barrier and be metabolized to inorganic Hg, which is therefore present at low doses in the central nervous system (CNS) [1, 2, 6].

Human populations throughout the world are chronically exposed to different species of Hg compounds [7–10]. Amazonian populations, for example, are exposed to Hg vapor due to the use of this metal in artisanal processes and small-scale gold mining [7, 8]. Additionally, because of the contaminated environment, populations living downstream of the mining areas or near dams (large-scale projects that may concentrate Hg in the environment) are exposed to both organic and inorganic mercury [8, 10]. The latter exposure is predominantly via the oral route due to contaminated food (mainly piscivorous fish) [11]. Once inside the human body, cells of CNS origin are able to accumulate organic mercury and partially transform it to inorganic mercury [6].

Although the additional effects of Hg intoxication (such as cardiovascular alterations and genotoxicity) are still being described, neurological symptoms are the main deleterious consequences of intoxication by this metal. These include alterations of motor coordination, progressive deterioration of visual and tactile senses, and paralysis, among others. Recent studies in both animals and humans also point to altered cognitive function, especially in memory and learning performance, caused by Hg exposure [12, 13].

The hippocampus is responsible for consolidating two types of memory, storage and recall of events. One of the memory types is aversive memory, related to emotions and reactions of fear and alertness, presenting the hippocampus and amygdala as regulatory areas [14]. The biochemical events occur at neural transmission routes, in which the hippocampus represents an area of huge importance in cognitive and behavioral analysis [14].

In the hippocampus, previous studies verified the presence of increased levels of Hg in the neural parenchyma after chronic exposure to HgCl_2_ [15], showing that Hg presents less tropism for the hippocampus when compared to the motor cortex. However, the tropism was sufficient to provoke a mnemonic dysfunction.

Based on this, we selected the cerebral hippocampal area for other analyses, aiming to study whether chronic exposure to inorganic Hg is capable of generating cognitive alterations and/or biochemical modulation, tissue damage, and cell death in the hippocampus of adult rats.

## 2. Materials and Methods

### 2.1. Ethics Statement

Experiments followed the protocol approved by the Ethics Committee on the use of animals (CEUA, Federal University of Pará, Protocol BIO139-13). They are in accordance with the NIH Guide for the Care and Use of Laboratory Animals and national law for laboratory experimentation [16].

### 2.2. Animals and Experimental Groups

Male Wistar rats (*n* = 56; 90 days old) from the Federal University of Pará (UFPA) animal facility were kept in collective cages (five animals per cage). The climate and light-controlled room was provided via a 12 h reverse light/dark cycle (lights on 7:00 a.m.), and animals received food and water ad libitum. Distilled water or HgCl_2_ (0.375 mg/kg/day; *n* = 20 per group) was orally administered by gavage over a period of 45 days, according to a procedure previously described by Teixeira et al. [15]. Animals were weighed weekly for HgCl_2_ dose adjustment.

### 2.3. Behavioral Assay: Step-Down Inhibitory Avoidance

After 24 h of HgCl_2_ or distilled water administration, animals (*n* = 14 per group) were taken to the behavioral test room for 1 h (acclimation), with controlled noise levels and illumination (12 lux). The step-down inhibitory avoidance apparatus (EP104R, Insight, Brazil) consists of an acrylic box (50 × 25 × 25 cm) with parallel stainless steel bars (1 mm in diameter), a floor connected to an electrical stimulator, and a secure platform (7 cm wide × 2.5 cm high) situated against the left wall.

Briefly, the animals were placed in the platform and were free to explore the apparatus for 3 minutes in a habituation session. Twenty-four hours later, the training session was conducted in which each animal was reintroduced to the secure place and the latency of stepping down onto the floor with all four paws was recorded (cut-off 180 s). Immediately after the animal step-down response onto the grid floor, a foot shock of 0.4 mA for 1 s was performed. In the sequence, the rats were removed from the inhibitory avoidance equipment, and after 1.5 h and 24 h, they were subjected to the short- and long-term memory test sessions, respectively [17].

### 2.4. Mercury Levels in the Hippocampus

To validate the exposure to HgCl_2_ and tissue levels compatible with the dose/time of administration, after the behavioral test, the animals (*n* = 7 per group) were euthanized and each hippocampus sample was weighed (0.5 g maximum of wet weight) in a sample digestion bottle. Then, 1 mL of distilled water, 2 mL of nitric acid-perchloric acid with HNO_3_-HClO_4_ (equal proportions), and 5 mL of sulfuric acid (H_2_SO_4_) were sequentially added, followed by heat treatment on a hot plate (200–230°C) for 30 min. Total Hg content in the samples was estimated by wet digestion, reduction, and cold vapor atomic absorption spectrometry (CVAAS) (semi-automated mercury analyzer, model Hg-201, Sanso Seisakusho Co. Ltd., Tokyo, Japan). The circulation-open airflow system was performed as previously described by Suzuki et al. [18]. The detection limit of the equipment for total Hg determination was 0.001 mg·kg^−1^, and the limit of quantification was 0.010 mg·kg^−1^. The results of the analyses of the samples were expressed in *μ*g/g. The obtained data were tabulated and later submitted to the inferential statistical treatment. The detailed methodology of this analysis is described in a previously published work [15].

### 2.5. Oxidative Stress

For this analysis, seven animals per group were euthanized by cervical dislocation. The hippocampus was collected and processed for biochemical assays. These animals were not submitted to the inhibitory avoidance equipment. The parameters evaluated were antioxidant capacity against peroxyl radicals (ACAP), evaluated through reactive oxygen species (ROS) determination in tissue samples treated or not with a peroxyl radical generator, by fluorimeter [19]; lipid peroxidation (LPO, using malondialdehyde—MDA—as an indicator); and nitrite levels (an indirect marker of nitric oxide production) by spectrophotometry, as previously described [20, 21]. For total protein evaluation, the method proposed by Bradford was used [22]. The results were expressed as percentages of the control groups.

### 2.6. Assessment and Quantification of Cytotoxicity and Apoptosis

In these assessments, animals (*n* = 7 per group) were euthanized by cervical dislocation and the hippocampus was dissected and treated with collagenase at a concentration of 2 mg/mL and 4 mg/mL and stored at 37°C for 20 minutes and 40 minutes, respectively, in order to dissociate the tissues (these animals were not submitted to inhibitory avoidance equipment). Thereafter, 100 mL of solution containing the isolated cells was added to 96-well microplates with 100 *μ*L of the CytoTox-Glo™ Cytotoxicity Assay. This assay uses a luminogenic peptide substrate to measure dead-cell protease activity or Caspase-Glo® 3/7 Assay Systems, a luminescent assay to measure caspase-3/7 activities (Promega, The Netherlands). Readings were performed in a GloMax® (Promega, The Netherlands) according to the manufacturer's recommendations. The quantification results were expressed as percentages of relative fluorescence units (RFU) or relative light units (RLU) relative to the control group, for cytotoxicity and apoptosis, respectively.

### 2.7. Histological Evaluation

#### 2.7.1. Perfusion and Histological Procedures

After behavioral assays, the remaining animals (*n* = 7 per group) were deeply anesthetized with ketamine hydrochloride (90 mg/kg, i.p.) and xylazine hydrochloride (10 mg/kg, i.p.), and transcardially perfused with heparinized 0.9% saline solution followed by 4% paraformaldehyde in 0.2 M phosphate buffer. Surgical manipulation was performed only after both the corneal and the paw withdraw reflexes were abolished. Brains were removed from the skull and postfixed for 6 h in Bouin solution. After postfixation, the brain tissue was embedded in paraplast (Monoject Scientific, Athy, Ireland) and sectioned on a microtome at 7 *μ*m thick and mounted in silanized slides. The coronal sections containing the anterior hippocampus were located at −3.60 mm posterior to Bregma [23].

#### 2.7.2. Immunohistochemistry

Sections of 7 *μ*m were submitted to immunohistochemistry analysis. The immunohistochemical procedures were described in our previous investigations [23, 24]. Briefly, the slides were dewaxed in xylol and hydrated at increasing concentrations of ethanol. Antigenic recovery was performed with citrate buffer at pH 6.0. In order to improve labeling intensity, sections were treated with 0.2 M boric acid (pH 9.0), previously heated to 65°C for 25 min. The temperature was maintained constant over the treatment period. Sections were kept at room temperature for 20 min to decrease the temperature and incubated under constant agitation in a 1% hydrogen peroxide solution in methanol for 20 min. The sections were rinsed in 0.05% PBS/Tween (Sigma Company, USA) solution for 5 min three times and incubated with 10% normal horse serum (NeuN) and goat serum (GFAP) in PBS for 1 h. Without further rinsing, sections were incubated overnight with the primary antibody in PBS, NeuN (1 : 500, Miliporere, USA) and GFAP (1 : 1000, Sigma, USA), rinsed in PBS/Tween solution for 5 min (3 times), and incubated with biotinylated horse anti-mouse (NeuN antibody) and goat anti-rabbit (GFAP), secondary antibodies (Vector Laboratories, USA) diluted at 1 : 500 in PBS for 2 h. As a negative control, normal serum, rather than primary antibody, was used in some sections.

Sections were rinsed again for 5 min (three times) and incubated in the avidin-biotin-peroxidase complex (ABC Kit, Vector Laboratories, USA) for 2 h. Sections were rinsed four times (5 min each) and revealed with diaminobenzidine (DAB). After the DAB reaction, sections were rinsed twice (5 min each) in 0.1 M PB, dehydrated, and cover-slipped with Entellan (Merck, Germany). For more details of this methodology, see [25, 26].

#### 2.7.3. Morphometric Analyses

**Figure 1:**
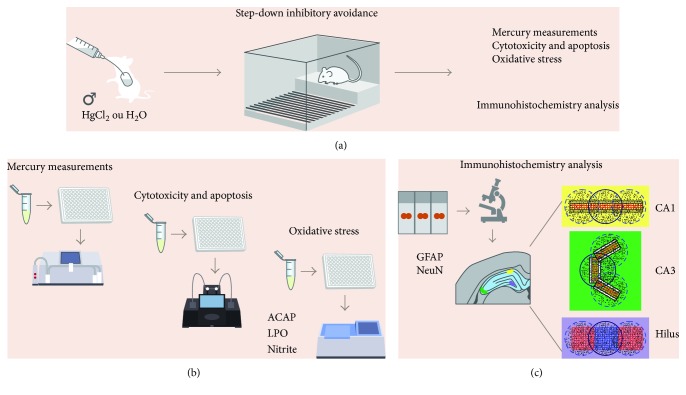
Sample description and experimental stages. Description of the sample and model of exposure to HgCl_2_; step-down inhibitory avoidance test; division of experimental groups and animal destinations for each stage of analysis (a); total Hg measurement assay, evaluation and quantification of cytotoxicity and apoptosis, and oxidative balance assays (b); immunohistochemistry and morphometric analysis (c).

For this quantification, a graticule (1 mm^2^) attached to the eyepiece (objective 40x, Nikon, Eclipse E200, USA) was used to count three fields per section and three sections per animal in CA1, CA3, and hilus, as described previously [17] and illustrated in Figure 1. NeuN-positive cells corresponding to mature neurons and GFAP-positive cells corresponding to astrocytes were counted. Illustrative images from all experimental groups were obtained with a digital camera (Moticam 2500, USA) attached to a microscope (Nikon, Eclipse 50i, USA).

### 2.8. Statistical Analysis

All values were tabulated and expressed as mean ± SEM (*n* = 14 animals per group in behavioral test and *n* = 7 per group in other analysis) and analyzed for normality using the Shapiro-Wilk test. To analyze body weight, we performed the one-way ANOVA test for repeated measures. Statistical comparisons between groups were performed using the Student's *t*-test. Values of *p* ≤ 0.05 were considered statistically significant. GraphPad Prism 5.0 (San Diego, CA, USA) software was used to perform statistical analyses.

The methods are summarized in Figure 1.

## 3. Results

### 3.1. Chronic HgCl_2_ Exposure Did Not Alter the Body Weight in Rats

**Figure 2:**
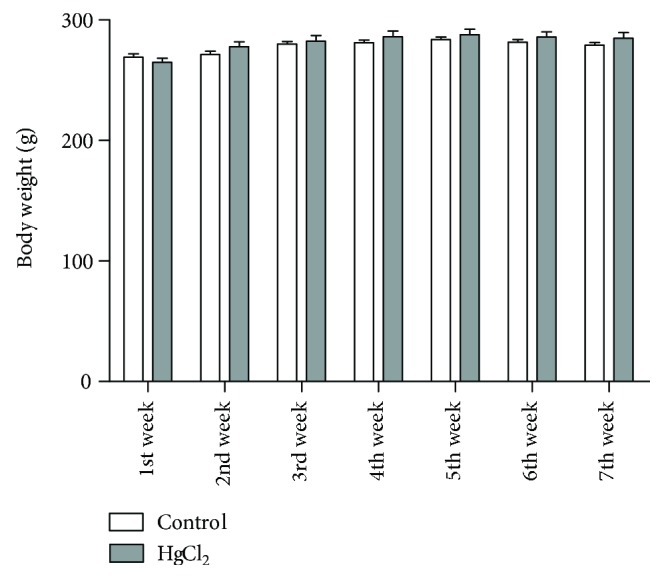
The effects of chronic exposure to HgCl_2_ on body weight of adult Wistar rats (g). Results are expressed as mean ± standard error after the two-way ANOVA analysis.

After 45 days of HgCl_2_ exposure, the body weight of control (278.1 ± 2.08 g) and HgCl_2_-exposed animals (281.3 ± 3.00 g) did not differ statistically (*p* = 0.38), as illustrated in Figure 2.

### 3.2. HgCl_2_ Exposure in Rats Displays Cognitive Deficits

**Figure 3:**
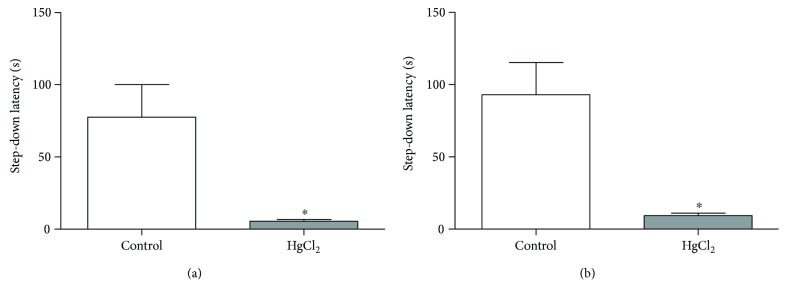
The effects of HgCl_2_ exposure (0.375 mg/kg/day) for 45 days on the short- and long-term memory of male Wistar rats evaluated in step-down inhibitory avoidance. The results are expressed as the mean ± SEM of the (a) latency to step down in seconds (1.5 h) and (b) latency to step down in seconds (24 h). ^∗^*p* < 0.05 compared to the control group (Student's *t*-test).

Chronic exposure by inorganic Hg during adult life impairs both short- and long-term memory in the inhibitory avoidance task Figure 3. These results were represented by a reduction in the step-down latency after 1.5 h training session in the animals exposed to Hg (5.60 ± 1.10 s) when compared to control (77.60 ± 22.48 s; *p* < 0.0001), as well as after the 24 h training session, in which the exposed animals (9.30 ± 1.77 s) presented lower values when compared to control (93.11 ± 22.20 s; *p* < 0.0001).

### 3.3. Analysis of Total Hg Levels Revealed the Presence of Metal in the Hippocampus after Chronic Exposure to HgCl_2_ in Rats

**Figure 4:**
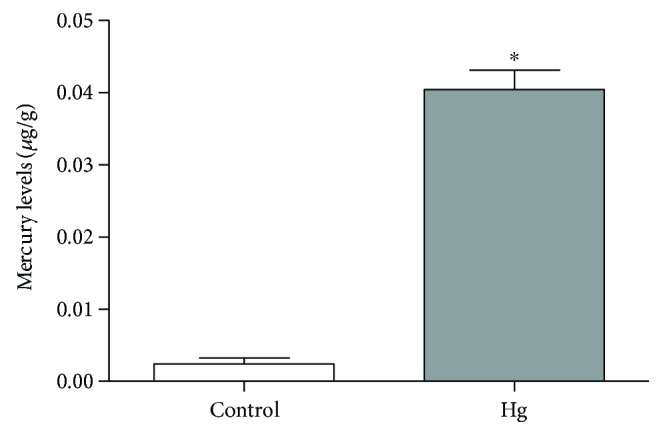
The effects of HgCl_2_ administration (0.375 mg/kg/day) for 45 days on the levels of Hg (*μ*g/g) in the hippocampus of male Wistar rats. The results are expressed as the mean ± SEM. ^∗^*p* < 0.05 compared to the control group (Student's *t*-test).

The Hg levels in the hippocampus of control animals (0.0024 ± 0.0008 *μ*g/g) differ significantly from exposed animals (0.0404 ± 0.0026 *μ*g/g; *p* < 0.0001).  Figure 4 displays the Hg levels in the hippocampus of rats after 45 days of intoxication, in *μ*g/g.

### 3.4. Chronic HgCl_2_ Exposure Reduces Antioxidant Capacity against Peroxyl Radicals and Increases Nitrite Levels and Lipid Peroxidation in the Hippocampus of Rats

**Figure 5:**
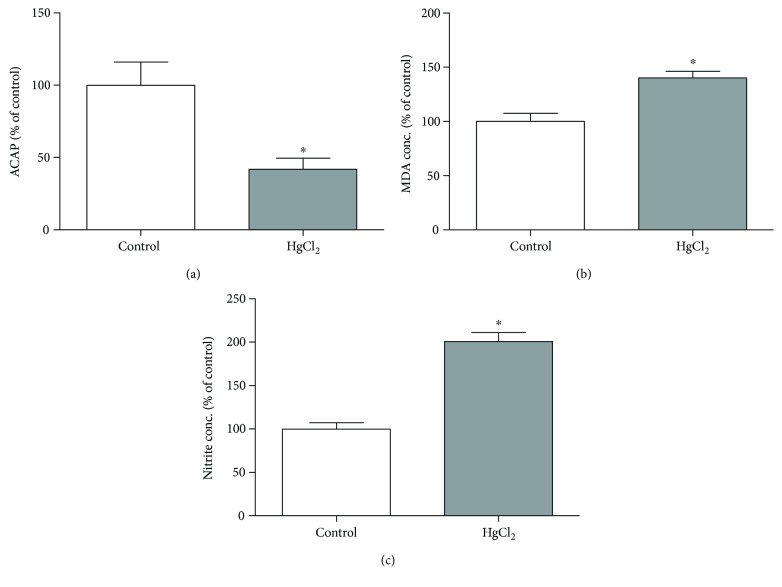
The effects of chronic exposure with HgCl_2_ on oxidative stress in the hippocampus of adult Wistar rats. The results are expressed as mean ± standard error of the (a) percentages of the fluorescence unit area difference of the generated curves of the same sample with and without ABAP in comparison to the control group; (b) percentages of milligram per malondialdehyde protein in relation to the control group; and (c) percentages of milligram per nitrite protein in relation to the control group. ^∗^*p* < 0.05 compared to the control group (Student's *t*-test).

The HgCl_2_ exposure decreases the antioxidant capacity against peroxyl radicals in the hippocampus of exposed animals (41.76 ± 7.68%) when compared to control animals (100 ± 16.01%; *p* = 0.0035; Figure 5(a)). Moreover, the chronic exposure with HgCl_2_ increased levels of prooxidant parameters, as evidenced by the increased levels of malondialdehyde (lipid peroxidation) in exposed animals (140.1 ± 6.08%), in comparison with the control group (100 ± 7.57%; *p* = 0.0028; Figure 5(b)). The same situation was registered in nitrite levels (HgCl_2_ = 200.9 ± 9.98%, control = 100 ± 7.25%; *p* = 0.0003; Figure 5(c)).

### 3.5. Chronic HgCl_2_ Exposure Led to Cytotoxicity and Cell Death by Apoptosis

**Figure 6:**
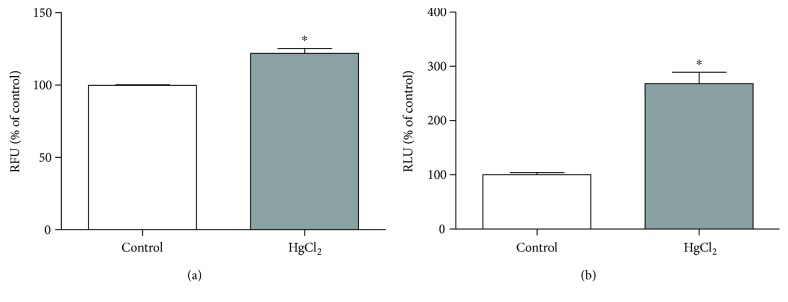
The effects of HgCl_2_ administration (0.375 mg/kg/day) for 45 days on cytotoxicity in the hippocampus of male Wistar rats evaluated in CytoTox-Glo reagent systems and apoptosis evaluated in ApoTox-Glo® reagent systems (Promega). The results are expressed as the mean ± SEM of the (a) relative fluorescence units (RFU) for cytotoxicity and (b) relative light units (RLU) for apoptosis. ^∗^*p* < 0.05 compared to the control group (Student's *t*-test).

The HgCl_2_ chronic exposure induced cytotoxicity in the hippocampus of exposed animals (121.9 ± 3.39% RFU) when compared to the control animals (100 ± 0.39% RFU; *p* = 0.0028; Figure 6(a)). Besides, the induction of apoptosis was increased in exposed animals (268.1 ± 21.00% RLU) when compared to control (100 ± 4.29% RLU; *p* < 0.0001; Figure 6(b)).

### 3.6. Chronic HgCl_2_ Exposure Reduces the Number of Astrocytes in the Hippocampus

**Figure 7:**
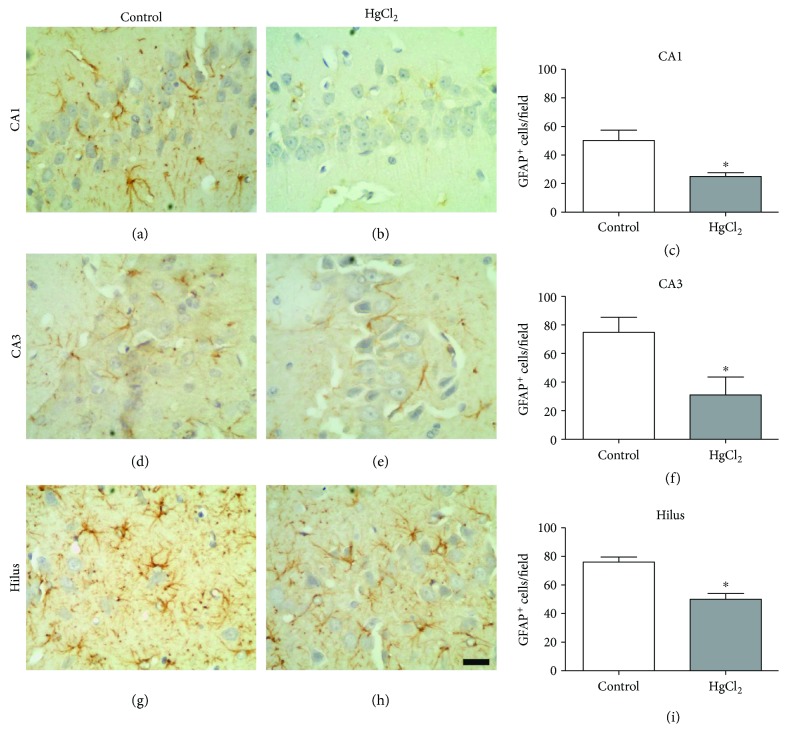
The effects of chronic exposure to HgCl_2_ on astrocyte density (GFAP^+^ cells) in the CA1 (a–c), CA3 (d–f), and hilus (g–i) of the hippocampus of adult Wistar rats. Results are expressed as mean ± standard error of the number of cells counted per field in each region (c, f, g). ^∗^*p* < 0.05 compared to the control group (Student's *t*-test). Scale bar: 20 *μ*m.

The GFAP^+^ cells in the hippocampus were affected by chronic HgCl_2_ exposure, as indicated by a significant reduction in cell density in all of the regions evaluated: CA1 (control 50.20 ± 7.29 cells/field; HgCl_2_ = 25.00 ± 2.64 cells/field; *p* = 0.043), CA3 (control 74.75 ± 10.64 cells/field; HgCl_2_ = 31.00 ± 12.50 cells/field; *p* = 0.044), and hilus (control 76.00 ± 3.50 cells/field; HgCl_2_ = 50.00 ± 4.04 cells/field; *p* = 0.003) (Figure 7).

### 3.7. Chronic HgCl_2_ Exposure Induces Neuronal Loss in the Hippocampus

**Figure 8:**
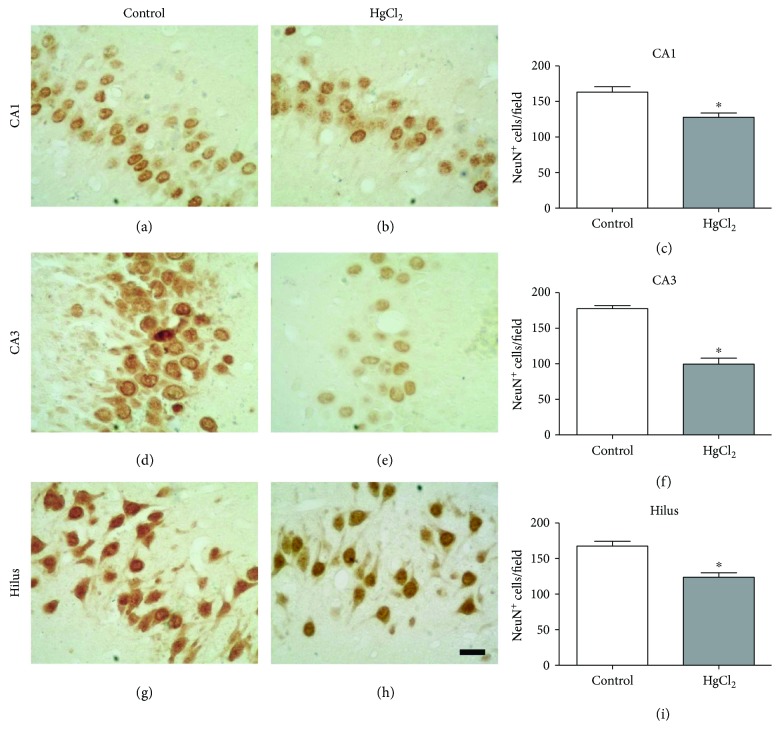
The effects of chronic exposure to HgCl_2_ on neuron density (NeuN^+^ cells) in the CA1 (a–c), CA3 (d–f), and hilus (g–i) of the hippocampus of adult Wistar rats. Results are expressed as mean ± standard error of the number of cells counted per field in each region (c, f, g). ^∗^*p* < 0.05 compared to the control group (Student's *t*-test). Scale bar: 20 *μ*m.

The quantification for NeuN^+^ cells showed that chronic HgCl_2_ exposure induced a significant neuronal loss in three of the analyzed regions: CA1 (control 163.3 ± 7.43 cells/field; HgCl_2_ = 127.8 ± 5.97 cells/field; *p* = 0.0070), CA3 (control 177.5 ± 4.48 cells/field; HgCl_2_ = 99.60 ± 8.38 cells/field; *p* = 0.0001), and hilus (control 167.5 ± 6.71 cells/field; HgCl_2_ = 123.8 ± 6.25 cells/field; *p* = 0.0021), as illustrated in Figure 8.

## 4. Discussion

This study shows that long-term chronic exposure to HgCl_2_ in adult rats promotes cognitive impairment, triggering oxidative stress and cell death in the hippocampus. This metal presents a low liposolubility, which disfavors its passage through the blood-brain barrier, especially in adults, with a few investigations described in the literature about the effects in the CNS [1, 2]. Unfortunately, scarce information is available about inorganic mercury burden in chronically exposed populations because epidemiological studies usually analyzed the total mercury content and not that of the different mercury species [8].

Even in this scenario, we chose to administer HgCl_2_ by intragastric gavage based on the fact that it represents an important form of intoxication by this metal [27, 28], reaching the systemic circulation and possibly crossing the blood-brain barrier and elevating the levels of Hg in neural parenchyma [15].

Models of chronic exposure to inorganic mercury are relatively uncommon in the literature, although the oral route was already shown to be an important route of chronic exposure to inorganic mercury [10, 11, 29]. Exposure to 0.8 mg/kg of inorganic mercury in a similar model did not cause obvious symptoms of toxicity in both pregnant rats and their pups (no movement disorder for adults and average body weight of newborns no less than 85% of controls). In this study, we used less than a half of that dose, characterizing an exposure to a relatively low concentration.

In this study, the analysis of total Hg levels revealed the presence of Hg in the hippocampus of chronically exposed rats to HgCl_2_, demonstrating that, despite its low liposolubility, inorganic Hg crossed the blood-brain barrier and deposited in the nervous tissue, even at relatively low oral doses. This fact indicates the possibility of exposure to HgCl_2_ during a prolonged period causing neurotoxic effects.

Hence, the Hg levels found were able to promote deficits in the short- and long-term memory tasks, evidenced by the inhibitory avoidance paradigm. In this trial, the animals were induced to learn and memorize an event through a noxious stimulus (electric shock). This test contains elements which cause a conflict in the motor response initiated by the animal, which previously held a noxious stimulus (walking on the grid and receiving an electric shock) [30].

Mello-Carpes et al. [31] have reported that occupational doses of HgCl_2_ after 60 days of exposure display deficits on the long-term memory in rats, evaluated 24 h after the training session. Our study went beyond such results, in which we investigated the short-term memory in the same paradigm. It is important to consider that the inhibitory avoidance test is an essential behavioral assay to evaluate memory function connected to the hippocampus [24, 31, 32].

Inhibitory avoidance memory, a type of fear memory, is formed in the hippocampus, with involvement of the basolateral amygdala [33]. In the hippocampus, especially in the dorsal hippocampus (posterior segment), the regions associated with a fear memory are dorsal CA1 and CA3 [34]. In this investigation, we analyzed the hippocampus as an anatomic region of choice for measurements of the levels of Hg, oxidative stress, and cell death.

Hg presents toxic effects in the CNS, resulting in changes in neurotransmission [34], deficiencies in neuronal differentiation [35], damage to cellular DNA [36, 37], changes in the cytoskeleton [35], variations in intracellular Ca^2+^ concentrations [38], and the production of reactive oxygen species (ROS) by configuring the mechanism of oxidative stress [39].

Thus, Hg-induced neurotoxicity is related to the overproduction of reactive oxygen and nitrogen species and/or a reduction of the antioxidant defense system [40–44]. It was demonstrated that chronic exposure to inorganic Hg promoted increased levels of malondialdehyde (MDA) and nitrites and decreased total antioxidant capacity by promoting oxidative stress in the hippocampus, similar to the results reported by Rizzetti et al., which evidenced the increase in MDA in the brain and plasma [45].

Our results of lipid peroxidation in the hippocampus after inorganic mercury exposure indicated that the neurotoxicity induced by Hg is due to the overproduction of free radicals and products of lipid peroxidation. This is confirmed by the concomitant decrease in total antioxidant capacity, by ACAP analysis. The ability of Hg to inhibit antioxidant enzymes such as glutathione peroxidase, glutathione reductase, superoxide dismutase, and catalase is already known because of its ability to form free and protein-free complexes, which may decrease or inhibit enzymatic activity [40, 43]. A recent study by our group revealed that Hg can modulate the ACAP of salivary glands, as it is a sensitive parameter to mercurial exposure [27].

The levels of Hg present in the hippocampus, associated with decreased antioxidant capacity and increased prooxidant factors, led us to evaluate the presence of cell death caused by cytotoxicity and the induction of apoptosis in the hippocampus. The chronic exposure to inorganic Hg in our model promoted cytotoxicity and apoptosis induction.

Similar results have been described based on *in vitro* studies, in which the inorganic Hg in cultured astrocytes and neurons promoted cell death by cytotoxicity in both cell types. This process is not associated with apoptotic mechanisms, even after prolonged exposure to the metal [46]. However, in our research, we show that both mechanisms of cell death can be triggered and occur concurrently.

In an animal experimentation model at a dose of 0.4 mg/kg, cell death by apoptosis, triggered by inorganic Hg, may occur from acute exposure, with an increase in caspase-3 activation and oxidative stress [40]. This occurs by an increase in the level of lipid peroxidation and inhibition of nitrite and the enzymatic antioxidant system. This decrease in antioxidant treatment could be represented by the decrease in the activity of superoxide dismutase, catalase, glutathione peroxidase, and glutathione reductase in the CNS [40]. These events are involved in neurotoxicity induced by Hg in the brain areas, thus increasing cytosolic Ca^2+^ levels, and therefore acting in a mechanism of cell death, which involves necrosis and the induction of apoptosis [40, 47, 48]. From our results, we can infer that HgCl_2_ can induce the decrease in both cell density and cytotoxicity by apoptosis, in adult organisms, with the processes occurring simultaneously in the hippocampus.

Chronic exposure to HgCl_2_ promotes tissue changes in the CA1, CA3, and hilus hippocampal areas, revealed by our immunohistochemical analyses. GFAP is the main protein constituent of the astrocyte cytoskeleton, thus being an important tissue marker [49]. The reduction of immunostaining in GFAP^+^ cell populations due to the neurotoxicity of HgCl_2_ found in our work points to a disorder of the mnemonic neural processes, since these cells belonging to the glia are auxiliary to the neurons and act in metabolic processes, in synaptic transmission, and in local homeostasis [50]. Previous studies have reported the increased susceptibility of astrocytes to inorganic Hg toxicity compared to neurons [51, 52]. This fact agrees with the study of Allen et al. [53], which indicates that the neurotoxicity is due to a deficiency of the astrocytic activity in the maintenance of metabolic pathways and interactions with neurons.

The reduction of neuronal cells in the hippocampal areas analyzed demonstrated the occurrence of the events of cell death caused by chronic exposure to HgCl_2_. The mechanisms involved in this process may be cytotoxicity or apoptosis, which in turn can be triggered by oxidative stress, as demonstrated in this study. This is in agreement with the results of Lohren et al. [46] which proved the inorganic Hg cytotoxicity in astrocytes and neurons, even though the occurrence of apoptotic processes was not observed. Another factor associated with this reduction of neuronal cells may be a notable reduction in their replacement due to the reduction of neurogenesis and gliogenesis [54].

Thus, inorganic Hg reaches and is deposited in the hippocampus. This accumulation promotes the increase of nitric oxide and other ROS levels, affecting membrane lipids and causing lipid peroxidation. The necrosis and apoptosis processes kill cells, both neurons and astrocytes. Finally, the decrease in the number of cells is the main cause of cognitive changes. The results presented in this study indicate the need for social measures and environmental policies aimed at determining the problem of Hg intoxication in populations living in contaminated regions.

In conclusion, chronic exposure to inorganic Hg during adulthood promotes cognitive disorders related to decreased cell density in the hippocampus, oxidative stress, cytotoxicity, and apoptosis induction.
